# Review of Chinese species of the leafhopper genus *Scaphoidella* Vilbaste, 1968 (Hemiptera, Cicadellidae, Deltocephalinae), with description of a new species

**DOI:** 10.3897/zookeys.491.8905

**Published:** 2015-03-26

**Authors:** Jichun Xing, Zizhong Li

**Affiliations:** 1 Institute of Entomology, Guizhou University; Guizhou Provincial Key Laboratory for Agricultural Pest Management of Mountainous Region; Special Key Laboratory for Development and Utilization of Insect Resources of Guizhou, Guiyang, Guizhou,P.R. China, 550025

**Keywords:** Homoptera, morphology, taxonomy, distribution, China

## Abstract

The Chinese leafhopper species of the genus *Scaphoidella* Vilbaste are reviewed, and one new species *Scaphoidella dietrichi* **sp. n.** is described and illustrated. Two species are recorded from China for the first time: *Scaphoidella clavatella* Dai & Dietrich, 2011 and *Scaphoidella zhangi* (Viraktamath & Mohan, 2004). A key based on the male genitalia is given to distinguish Chinese species of this genus and a map provided their geographic distribution. The type specimens of the new species is deposited in the Institute of Entomology, Guizhou University, Guiyang, China (GUGC).

## Introduction

The leafhopper genus *Scaphoidella* was established by Vilbaste (1968) for *Scaphoidella arboricola* Vilbaste from the Maritime Territory of Russia. It belongs to the tribe Scaphoideini of subfamily Deltocephalinae (Zahniser & Dietrich, 2013). Recently, Dai and Dietrich (2011) reviewed this genus and described ten new species from Thailand and Vietnam and at the same time, two new combinations, *Scaphoidella zhangi* (Viraktamath & Mohan, 2004), and *Scaphoidella punctulata* (Melichar, 1903) were proposed (both previously placed in the genus *Scaphoideus*), the species *Scaphoidella datianensis* Xing, Dai & Li, 2008 was placed in the genus *Monobazus*, and *Scaphoidella transversa* Li & Xing, 2009 was excluded from *Scaphoidella* and treated as a *species incertae sedis*. The species *Scaphoidella denticlestyla* Xing & Li, 2010 (see Chen, Li and Jin 2010) and *Scaphoidella brevissima* Dai, Xing & Li (2011) in *Scaphoidella* (not listed in the checklist of Dai and Dietrich (2011)) are discussed below, bringing the total of known species to 20, including eight from China.

In this paper, a new species: *Scaphoidella dietrichi* sp. n. is described and illustrated from Yunnan Province, China, and *Scaphoidella clavatella* Dai & Dietrich, 2011 and *Scaphoidella zhangi* (Viraktamath & Mohan, 2004) are recorded from China for the first time. The type specimens of the new species is deposited in the Institute of Entomology, Guizhou University, Guiyang, China (GUGC). The genus *Scaphoidella* now contains 21 species including 11 from China. A key is given to separate the Chinese species.

## Material and methods

Terminology of morphological and genital characters follow Zhang and Dai (2006) and Dai and Dietrich (2011). Male specimens were used for the description and illustration. External morphology was observed under a stereoscopic microscope and characters were measured with an ocular micrometer. Color pictures for adult habitus were obtained by KEYENCE VHX-1000 system. The genital segments of the examined specimens were macerated in 10% NaOH and drawn from preparations in glycerin jelly using a Leica MZ 12.5 stereomicroscope. Illustrations were scanned with Canon CanoScan LiDE 200 and imported into Adobe Photoshop 8 for labeling and plate composition.

## Taxonomy

### Scaphoidella

Taxon classificationAnimaliaHemipteraCicadellidae

Vilbaste

Scaphoidella Vilbaste, 1968: 133; Zhang and Dai 2006: 841; Li, Dai and Xing 2011: 199; Dai and Dietrich 2011: 458.

#### Type species.

*Scaphoidella arboricola* Vilbaste, 1968.

For the relationship and diagnosis of *Scaphoidella* Vilbaste see Dai and Dietrich (2011: 458).

#### Distribution.

Oriental Region and Palaearctic Region (see Discussion).

#### Checklist of species of *Scaphoidella*

*Scaphoidella acaudata* Zhang & Dai, 2006 Distribution: China (Yunnan, Guizhou).

*Scaphoidella arboricola* Vilbaste, 1968 Distribution: Russia (Maritime Territory); China (Zhejiang, Henan).

*Scaphoidella bifurcata* Dai & Dietrich, 2011 Distribution: Thailand (Chaiyaphum, Ubon Ratchathani, Phetchabun).

*Scaphoidella brevissima* Dai, Xing & Li, 2011 Distribution: China (Henan).

*Scaphoidella clavatella* Dai & Dietrich, 2011 Distribution: Thailand (Loei); China (Guangxi, Yunnan).

*Scaphoidella cornuta* Dai & Dietrich, 2011 Distribution: Thailand (Loei, Phetchabun).

*Scaphoidella coronoida* Dai & Dietrich, 2011 Distribution: Thailand (Chiang Mai, Loei).

*Scaphoidella denticlestyla* Xing & Li, 2010 Distribution: China (Guizhou).

*Scaphoidella dietrichi* Xing & Li, sp. n. Distribution: China (Yunnan).

*Scaphoidella digitatus* Dai & Dietrich, 2011 Distribution: Thailand (Ubon Ratchathani, Khonkaen).

*Scaphoidella dimidiatus* Dai & Dietrich, 2011 Distribution: Thailand (Chaiyaphum, Loei, Phetchabun).

*Scaphoidella dongnaiensis* Dai & Dietrich, 2011 Distribution: Vietnam (Dongnai).

*Scaphoidella flangenella* Dai & Dietrich, 2011 Distribution: Thailand (Ubon Ratchathani).

*Scaphoidella lamella* Dai & Dietrich, 2011 Distribution: Thailand (Loei, Phetchabun, Phitsanulok, Sakon Nakhon, Ubon Ratchathani).

*Scaphoidella punctulata* (Melichar, 1903) Distribution: Sri Lanka.

*Scaphoidella stenopaea* Anufriev, 1977 Distribution: Russia (Amur Province, Maritime Territory); China (Shaanxi, Shandong, Heilongjiang, Liaoning, Gansu, Inner Mongolia, Hebei, Shanxi).

*Scaphoidella undosa* Zhang & Dai, 2006 Distribution: China (Henan, Hunan, Jiangxi, Hubei, Guizhou, Zhejiang, Anhui).

*Scaphoidella unihamata* (Li & Kuoh, 1993) Distribution: China (Zhejiang, Hunan, Fujian, Guangxi).

*Scaphoidella viraktamathi* Dai & Dietrich, 2011 Distribution: Thailand (Phetchabun, Sakon Nakhon).

*Scaphoidella wideaedeaga* (Wang & Li, 2004) Distribution: China (Yunnan, Xizang); Thailand (Loei).

*Scaphoidella zhangi* (Viraktamath & Mohan, 2004) Distribution: India (Meghalaya, West Bengal); Thailand (Loei); China (Guizhou).

#### Key to species (males) of *Scaphoidella* from China

**Table:** 

1	Pygefer side with conspicuous spine on dorsal margin (Figs 19, 37)	**2**
–	Pygefer side without spine on dorsal margin	**3**
2	Pygofer side with caudal margin round, without spine (Fig. 19)	***Scaphoidella clavatella***
–	Pygofer side with ventrally directed spine on caudal margin (Fig. 37)	***Scaphoidella zhangi***
3	Subgenital plate with lateral macrosetae arranged irregularly (Zhang and Dai 2006: Figs 5, 15)	**4**
–	Subgenital plate with lateral macrosetae in single row	**5**
4	ygofer process long; subgenital plate tapered apically; basal processes of aedeagus extending to near apex of shaft (Zhang and Dai 2006: Figs 4–6)	***Scaphoidella undosa***
–	Pygofer process short; subgenital plate rounded apically; basal processes of aedeagus extending beyond shaft (Zhang and Dai 2006: Figs 14–16)	***Scaphoidella arboricola***
5	Pygofer side with caudal process (Fig. 13; Zhang and Dai 2006: Figs 33, 44)	**6**
–	Pygofer side without caudal process	**8**
6	Apex of aedeagal shaft with pair of lateral processes; preatrium short (Zhang and Dai 2006: Figs 28, 29)	***Scaphoidella stenopaea***
–	Apex of aedeagal shaft without processes; preatrium very long (Fig. 17; Zhang and Dai 2006: Fig. 37)	**7**
7	Style apical process moderately long (Fig. 18)	***Scaphoidella brevissima***
–	Style apical process very long (Zhang and Dai 2006: Fig. 39)	***Scaphoidella unihamata***
8	Apical margin of aedeagal shaft with many small spines on both sides (Figs 34, 35)	***Scaphoidella dietrichi* sp. n.**
–	Aedeagal shaft without small spinose processes	**9**
9	Aedeagal shaft in lateral view distinctly broadened near midlength; stem of connective nearly 1/3 length of arms (Zhang and Dai 2006: Figs 47, 50)	***Scaphoidella wideaedeaga***
–	Aedeagal shaft in lateral view slender and not broadened near midlength; stem of connective and arms of approximately equal length (Figs 28, 29; Zhang and Dai 2006: Figs 57, 58)	**10**
10	Preatrium of aedeagus very long; style apical process with teeth (Figs 29, 30)	***Scaphoidella denticlestyla***
–	Preatrium of aedeagus short; style apical process without teeth (Zhang and Dai 2006: Figs 55, 57)	***Scaphoidella acaudata***

### Chinese *Scaphoidella* species

#### Scaphoidella acaudata

Taxon classificationAnimaliaHemipteraCicadellidae

Zhang & Dai, 2006

**Figures 1–8.**
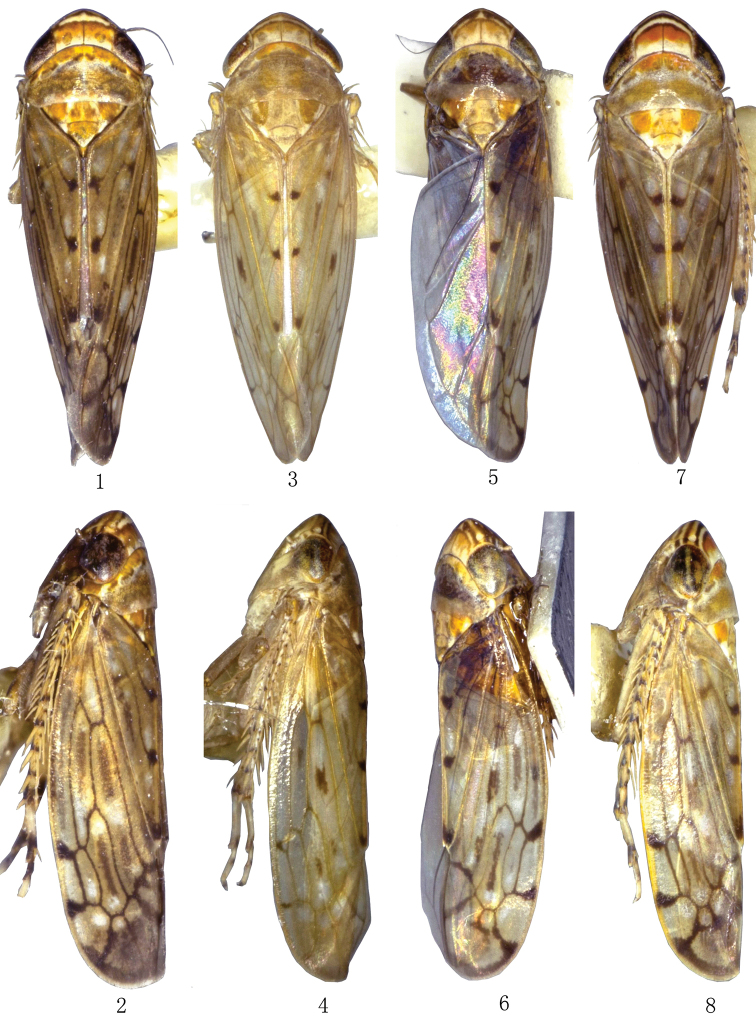
*Scaphoidella* species. **1, 2** *Scaphoidella brevissima* Dai, Xing & Li **1** ♂, dorsal view **2** ♂, lateral view **3, 4** *Scaphoidella clavatella* Dai & Dietrich **3** ♂, dorsal view **4** ♂, lateral view **5, 6** *Scaphoidella denticlestyla* Xing & Li **5** ♂, dorsal view **6** ♂, lateral view **7, 8** *Scaphoidella zhangi* (Viraktamath & Mohan) **7** ♂, dorsal view **8** ♂, lateral view.

Scaphoidella acaudata Zhang & Dai, 2006: 850, figs 51–58; Li, Dai and Xing 2011: 199, plate 5–194, figs 1–6.

##### Material examined.

1♂, China: Guizhou Prov., Bailidujuan, 18 October 2007, coll. Yujian Li (GUGC); 1♂, Yunnan Prov., Longling, Longxin, 10 June 2011, coll. Jiankun Long (GUGC).

##### Distribution.

China (Yunnan, Guizhou) (Fig. 43).

#### Scaphoidella arboricola

Taxon classificationAnimaliaHemipteraCicadellidae

Vilbaste, 1968

Scaphoidella arboricola Vilbaste, 1968: 133, plate 110, figs 1–8; Zhang and Dai 2006: 850, figs 1–10.

##### Distribution.

Russia (Maritime Territory); China (Zhejiang, Henan) (Fig. 43).

#### Scaphoidella brevissima

Taxon classificationAnimaliaHemipteraCicadellidae

Dai, Xing & Li, 2011

Figs 1–2 , 13–18

Scaphoidella brevissima Dai, Xing & Li, 2011: 1, figs 1–10.

##### Material examined.

China: 1♂ (Holotype), Henan Prov., Luanchuan County, Heyu, 19 August 2008, coll. Jichun Xing (GUGC); 1♂, Henan Prov., Xixia County, Taiping, 30 July 2010, coll. Hu Li and Zhihua Fang (GUGC).

##### Distribution.

China (Henan) (Fig. 43).

##### Note.

This species was described from China (Henan) based on the male holotype (GUGC).

**Figures 9–12.**
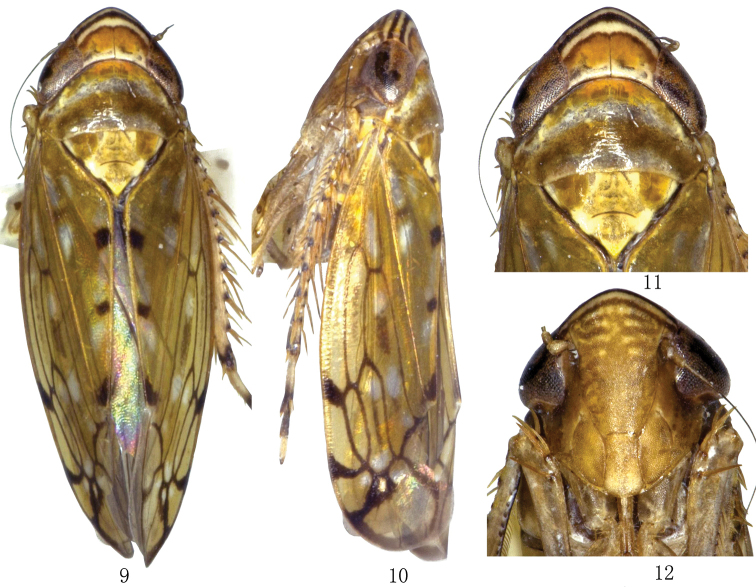
*Scaphoidella dietrichi* sp. n., ♂. **9** dorsal view **10** lateral view **11** head and thorax, dorsal view **12** face.

**Figures 13–18.**
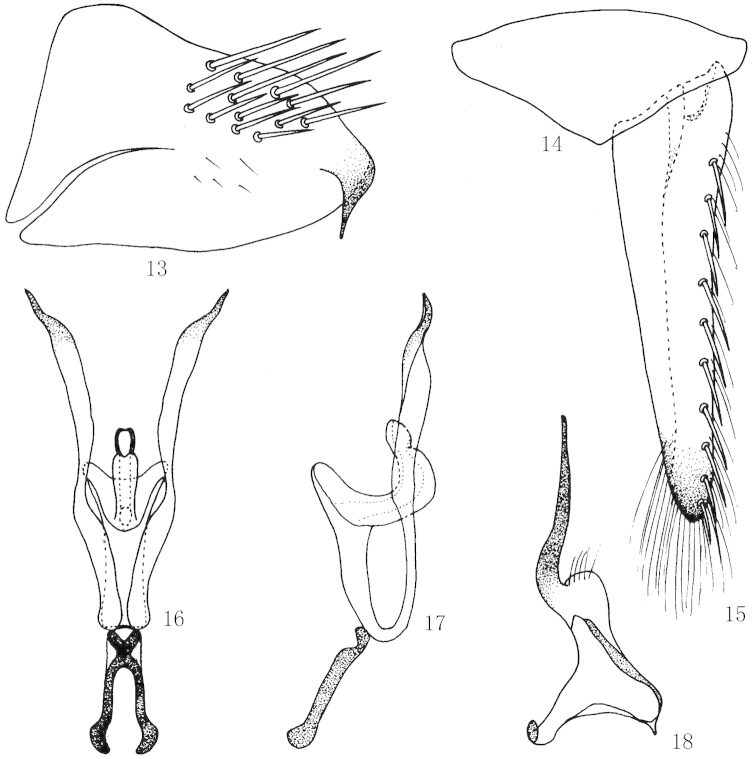
*Scaphoidella brevissima* Dai, Xing & Li, **13** Male pygofer side, lateral view **14** Valve, ventral view **15** Subgenital plates, ventral view **16** Aedeagus and connective, ventral view **17** Aedeagus and connective, lateral view **18** Style, dorsal view.

#### Scaphoidella clavatella

Taxon classificationAnimaliaHemipteraCicadellidae

Dai & Dietrich, 2011

Figs 3–4 , 19–24

Scaphoidella clavatella Dai & Dietrich, 2011: 468. figs 51–55.

##### Material examined.

1♂, China: Guangxi Autonomous Region, Daxin County, Detianpubu, 11 May 2012, coll. Zhihua Fan (GUGC); 1♂1♀, Yunnan Prov., Menghai, 13 July 2013, coll. Jichun Xing (GUGC).

##### Distribution.

Thailand (Loei); China (Guangxi, Yunnan) (Fig. 43).

##### Note.

This species was described from Thailand (Loei) based on two male specimens (QSBG and INHS). This species is here recorded from China for the first time.

**Figures 19–24.**
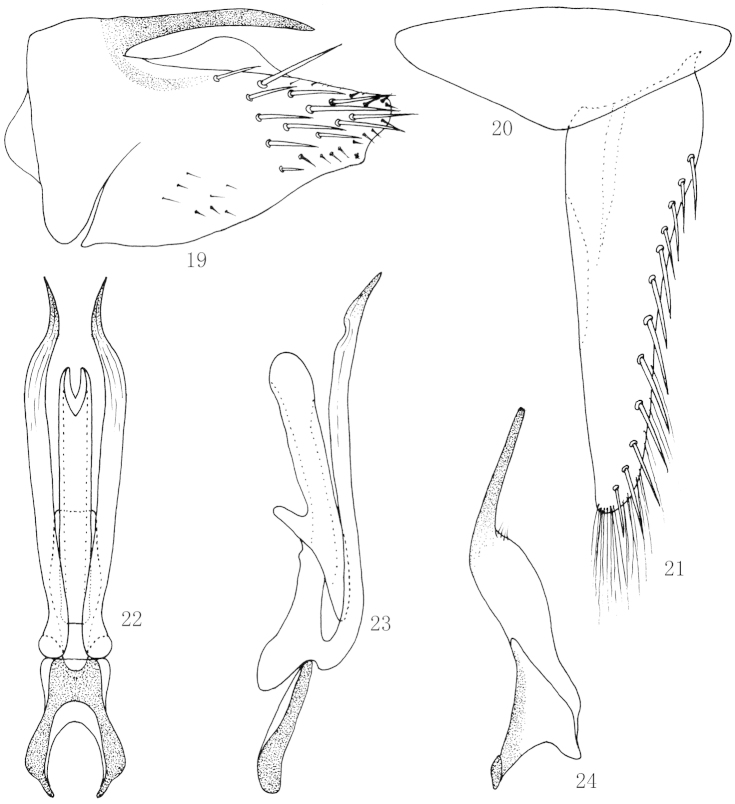
*Scaphoidella clavatella* Dai & Dietrich, **19** Male pygofer side, lateral view **20** Valve, ventral view **21** Subgenital plates, ventral view **22** Aedeagus and connective, ventral view **23** Aedeagus and connective, lateral view **24** Style, dorsal view.

#### Scaphoidella denticlestyla

Taxon classificationAnimaliaHemipteraCicadellidae

Xing & Li, 2010

Figs 5–6 , 25–30

Scaphoidella denticlestyla Xing & Li (in Chen et al.), 2010: 138, Figs 7–14; Li, Dai and Xing 2011: 200, plate 5–195, figs 1–8.

##### Material examined.

China: 1♂ (Holotype), Guizhou Prov., Mayanghe, Maojia, 5 October 2007, coll. Yujian Li (GUGC); 1♂, Guizhou Prov., Mayanghe, Maojia, 6 October 2007, coll. Qiongzhang Song (GUGC).

##### Distribution.

China (Guizhou) (Fig. 43).

##### Note.

This species was described from China (Guizhou) based on two male specimens deposited in GUGC. As the original figures of Xing & Li (in Chen et al. 2010 and Li et al. 2011) are not very perfect the male genitalia are redrawn here by the first author.

**Figures 25–30.**
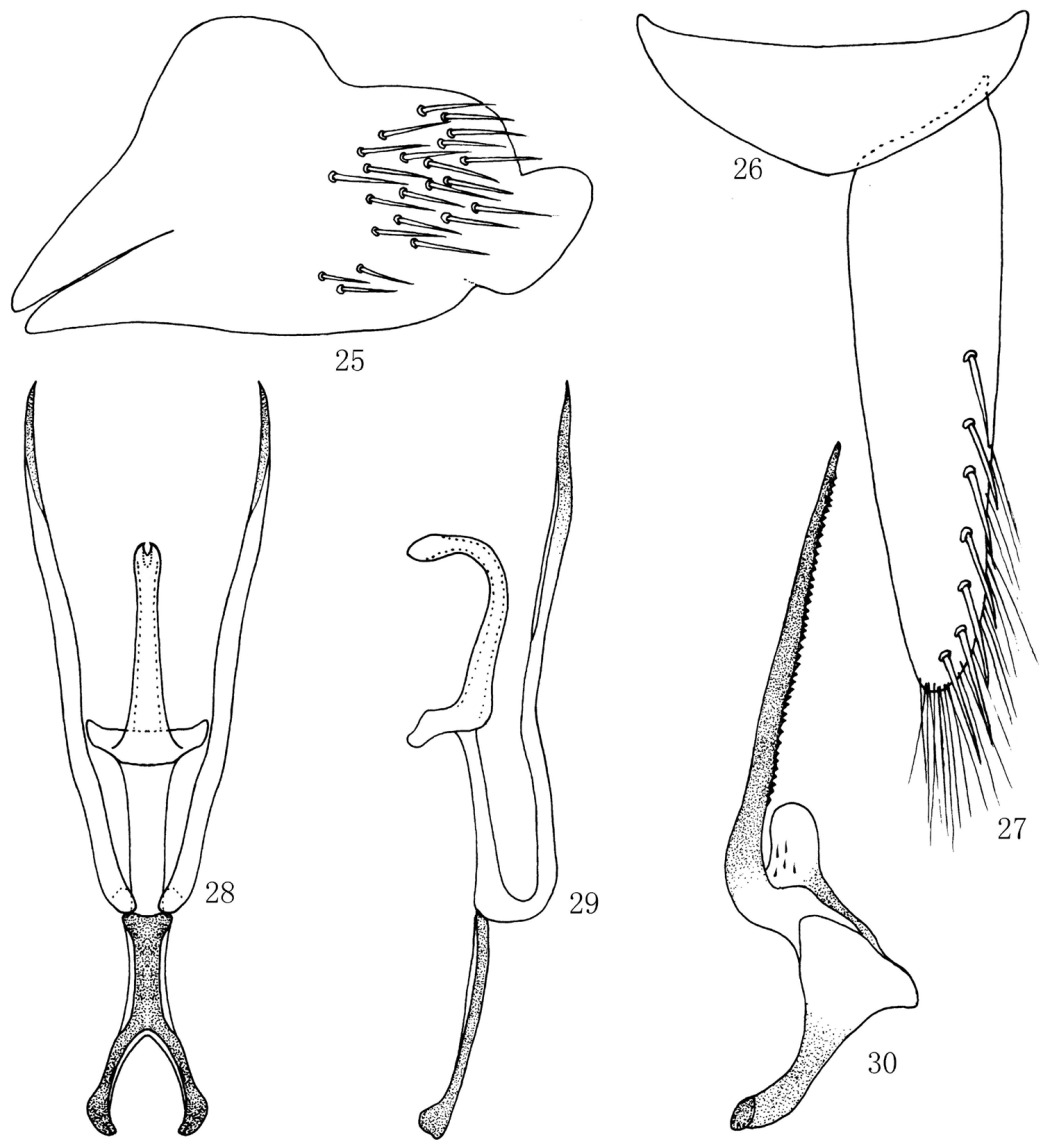
*Scaphoidella denticlestyla* Xing & Li, **25** Male pygofer side, lateral view **26** Valve, ventral view **27** Subgenital plates, ventral view **28** Aedeagus and connective, ventral view **29** Aedeagus and connective, lateral view **30** Style, dorsal view.

#### Scaphoidella dietrichi

Taxon classificationAnimaliaHemipteraCicadellidae

Xing & Li sp. n.

http://zoobank.org/414D9E83-6DF9-4F74-BECC-55886B131911

Figs 9–12 , 31–36

##### Description.

Body ochraceous. Head with piceous submargial band on anterior margin, one transverse arcuate band between eyes anteriorly, narrowly margined with piceous, orange red (Figs 9, 11). Face with thin, arcuate, piceous submarginal band (Fig. 12). Pronotum with anterior brown and posterior submarginal chocolate brown transverse bands (Fig. 11). Forewing ochraceous, with hyaline spots (Figs 9, 10).

Vertex shorter than pronotum, shorter medially than next to eye. Pronotum longer than scutellum (Fig. 11). External features as in generic description (see Dai and Dietrich 2011: 458).

**Figures 31–36.**
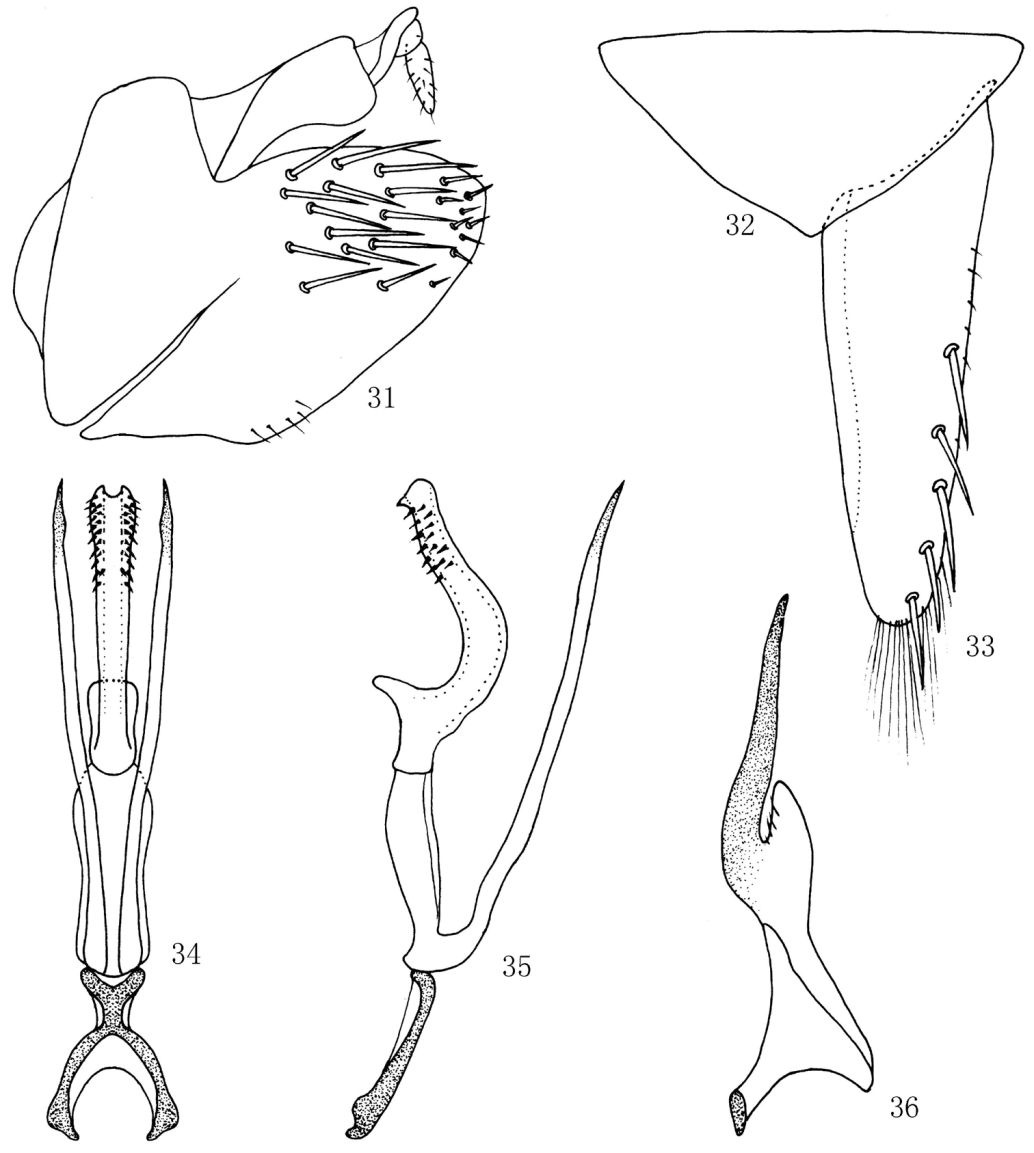
*Scaphoidella dietrichi* sp. n., **31** Male pygofer side, lateral view **32** Valve, ventral view **33** Subgenital plates, ventral view **34** Aedeagus and connective, ventral view **35** Aedeagus and connective, lateral view **36** Style, dorsal view.

*Male genitalia.* Pygofer in lateral aspect tapering posteriorly from midlength, with many short and long macrosetae dorsally, without caudal process (Fig. 31). Valve large, subtriangular (Fig. 32). Subgenital plate elongate, narrowing to rounded apex, uniseriate row of macrosetae along ventrolateral margin and additional hair-like setae at apex (Fig. 33). Aedeagal shaft curved dorsally, its apical margin with many small spines on both sides, gonopore apical, preatrium very long; basal processes slender, tapering apically, extended to near apex of aedeagal shaft (Figs 34, 35). Connective Y-shaped, articulated with aedeagus, its stem nearly 1/3 length of arms (Fig. 34). Style elongate, with prominent subapical lobe, apophysis slender and narrowed distally, equal to 1/2 length of style (Fig. 36).

##### Measurement.

Length (including tegmen): ♂, 4.4 mm.

##### Type material.

Holotype ♂, China: Yunnan Prov., Xishuangbanna, Menglun, 28 July 2012, coll. Weibin Zheng (GUGC).

##### Host.

Grasses.

##### Distribution.

China (Yunnan) (Fig. 43).

##### Diagnosis.

This species is similar to *Scaphoidella zhangi* (Viraktamath & Mohan, 2004), but can be distinguished from the latter by the male pygofer without caudal process and spine on dorsal margin, apical margin of aedeagal shaft with many small spinose processes on both sides, and aedeagal shaft curved dorsally.

##### Etymology.

This new species is named after Dr. C. H. Dietrich (INHS) in recognition of his good work on leafhoppers.

#### Scaphoidella stenopaea

Taxon classificationAnimaliaHemipteraCicadellidae

Anufriev, 1977

Scaphoidella stenopaea Anufriev, 1977: 213, figs 13–19; Zhang and Dai 2006: 847, figs 23–29; Li, Dai and Xing 2011: 201, plate 5-196, figs 1–6.

##### Material examined.

3♂♂4♀♀, China: Inner Mongolia Autonomous Region, Zhalantun, 26 August 1996, coll. Zizhong Li; 2♂♂3♀♀, Gansu Prov., Zhenyuan, 1 August 2007, coll. Wei Cao; 4♂♂2♀♀, Hebei Prov., Chengde, Wulingshan, 14 August 2010, coll. Lixia Xie; 2♂♂, Shanxi Prov., Lishan, Dahelinchang, 23 July 2012, coll. Jichun Xing. All GUGC.

##### Distribution.

Russia (Amur Province, Maritime Territory); China (Shaanxi, Shandong, Heilongjiang, Liaoning, Gansu, Inner Mongolia, Hebei, Shanxi) (Fig. 43).

#### Scaphoidella undosa

Taxon classificationAnimaliaHemipteraCicadellidae

Zhang & Dai, 2006

Scaphoidella undosa Zhang & Dai, 2006: 844, figs 11–22; Li, Dai and Xing 2011: 204, plate 5-199, figs 1–7.

##### Material examined.

1♂2♀♀, China: Guizhou Prov., Kuankuoshui, 24 August 2001, coll. Zizhong Li; 1♂, Guizhou Prov., Congjiang County, Yueliangshan, 20 July 2006, coll. Zaihua Yang; 1♂1♀, Zhejiang Prov., Tianmushan, 22 July 2009, light trap coll. Zehong Meng; 1♂, Guizhou Prov., Kuankuoshui, 11 August 2010, coll. Hu Li; 2♂♂1♀, Guizhou Prov., Kuankuoshui, 14 August 2010, coll. Jichun Xing; 2♂♂, Guizhou Prov., Kuankuoshui, 17 August 2010, coll. Hu Li and Zhihua Fan; 2♂♂, Anhui Prov., Jinzhai County, Tianma, 31 July 2013, coll. Bin Li. All GUGC.

##### Distribution.

China (Henan, Hunan, Jiangxi, Hubei, Guizhou, Zhejiang, Anhui) (Fig. 43).

#### Scaphoidella unihamata

Taxon classificationAnimaliaHemipteraCicadellidae

(Li & Kuoh, 1993)

Scaphoideus unihamatus Li & Kuoh, 1993: 39, figs 7–12.
Scaphoidella inermis Cai & He, 2001: 205, figs 89–96, synonymised by Zhang and Dai 2006: 848.
Scaphoidella unihamata (Li & Kuoh), comb. n. by Zhang and Dai 2006: 848, figs 30–40; Li, Dai and Xing 2011: 203, plate 5–198, figs 1–5.

##### Material examined.

China: 1♂ (Holotype), Fujian Prov., Sanming, 6 September 1978, coll. Zhonglin Ge; 2♂♂, Fujian Prov., Sanming, 6 September 1978, coll. Zhonglin Ge; 1♂, Guangxi Autonomous Region, Huaping, 19 May 2012, coll. Zhihua Fan. All GUGC.

##### Distribution.

China (Zhejiang, Hunan, Fujian, Guangxi) (Fig. 43).

#### Scaphoidella wideaedeaga

Taxon classificationAnimaliaHemipteraCicadellidae

(Wang & Li, 2004)

Scaphoideus wideaedeagus Wang & Li, 2004: 17, figs 14–19.
Scaphoidella wideaedeaga (Wang & Li), comb. n. by Zhang and Dai 2006: 849, figs 41–50; Li, Dai and Xing 2011: 205, plate 5-200, figs 1–6; Dai and Dietrich 2011: 472.

##### Material examined.

China: 1♂ (Holotype), Yunnan Prov., Tengchong, 4 July 2002, coll. Renhuai Dai; 1♂1♀, Yunnan Prov., Gaoligongshan, Baihualing, 14 June 2011, coll. Yujian Li; 1♂, Yunnan Prov., Ruili City, Nongdao, 15 July 2013, coll. Weicheng Yang; 1♂, Yunnan Prov., Gaoligongshan, Baihualing, 5 August 2013, coll. Zhihua Fan. All GUGC.

##### Distribution.

China (Yunnan, Xizang) (Fig. 43), Thailand (Loei).

#### Scaphoidella zhangi

Taxon classificationAnimaliaHemipteraCicadellidae

(Viraktamath & Mohan, 2004)

Figs 7–8 , 37–42

Scaphoideus zhangi Viraktamath & Mohan, 2004: 45, figs 218–227.
Scaphoidella zhangi comb. n. by Dai and Dietrich 2011: 471.

##### Material examined.

1♂, China: Guizhou Prov., Luodian County, Bamao, 20 October 2002, coll. Renhuai Dai (GUGC).

##### Distribution.

India (Meghalaya, West Bengal); Thailand (Loei); China (Guizhou) (Fig. 43).

##### Note.

This species is here recorded from China for the first time.

**Figures 37–42.**
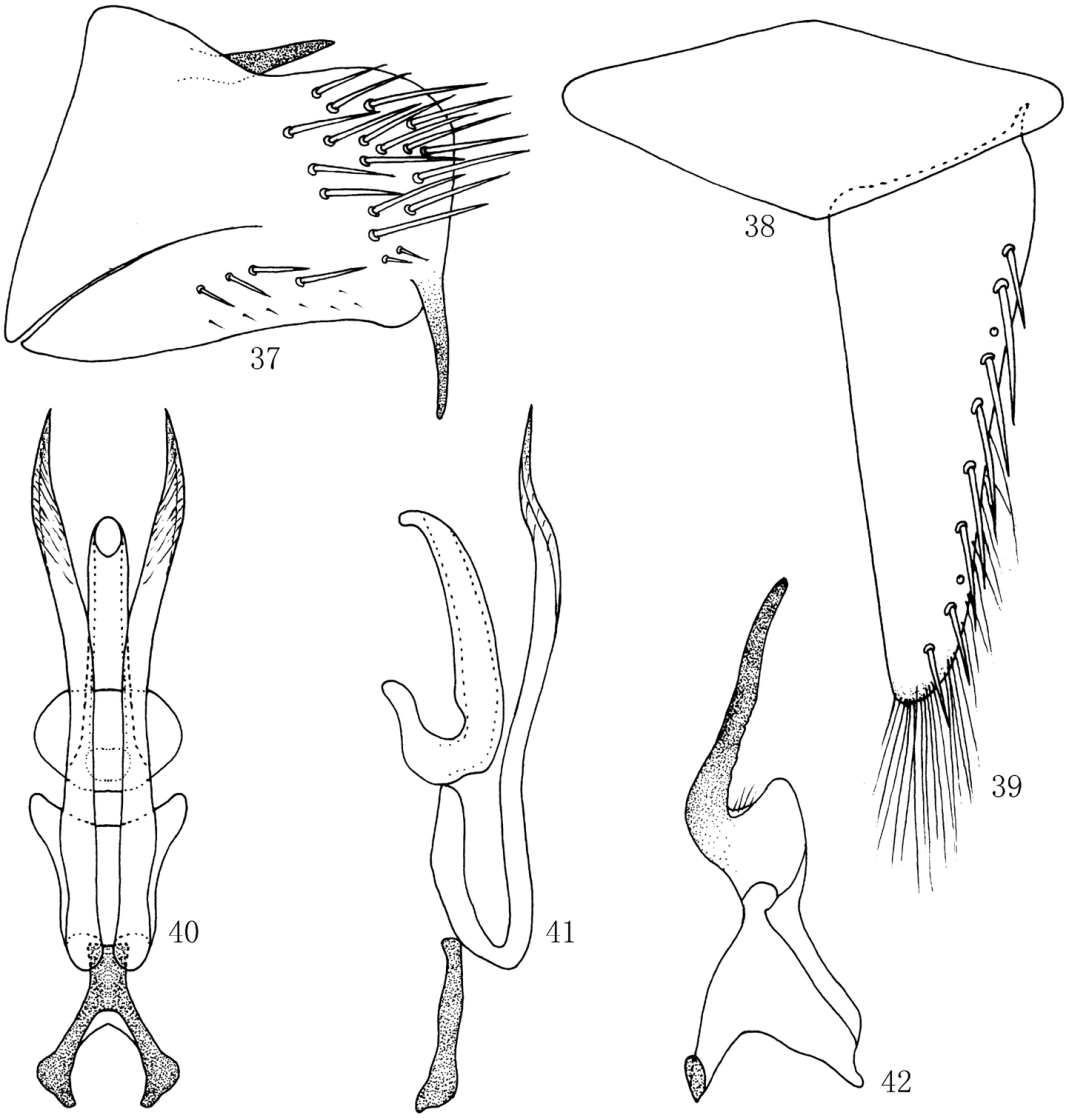
*Scaphoidella zhangi* (Viraktamath & Mohan), **37** Male pygofer side, lateral view **38** Valve, ventral view **39** Subgenital plates, ventral view **40** Aedeagus and connective, ventral view **41** Aedeagus and connective, lateral view **42** Style, dorsal view.

**Figure 43.**
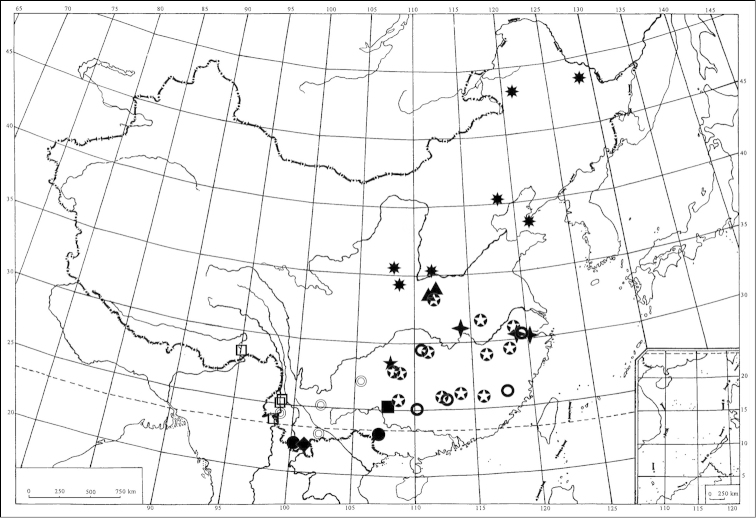
Geographic distribution of *Scaphoidella* species in China: *Scaphoidella acaudata* (◎); *Scaphoidella arboricola* (✦); *Scaphoidella brevissima* (▲); *Scaphoidella clavatella* (●); *Scaphoidella denticlestyla* (★); *Scaphoidella dietrichi* sp. n. (◆); *Scaphoidella stenopaea* (✴); *Scaphoidella undosa* (○); *Scaphoidella unihamata* (✪); *Scaphoidella wideaedeaga* (□); *Scaphoidella zhangi* (■).

## Discussion

Chinese species of *Scaphoidella* are mainly distributed in southern China (*Scaphoidella acaudata*, *Scaphoidella clavatella*, *Scaphoidella denticlestyla*, *Scaphoidella dietrichi*, *Scaphoidella unihamata*, and *Scaphoidella zhangi*) with *Scaphoidella brevissima* and *Scaphoidella stenopaea* distributed in the Palaearctic Region (northern China and also Inner Mongolia). The following Chinese species occur in both regions: *Scaphoidella arboricola*, *Scaphoidella undosa*, and *Scaphoidella wideaedeaga*. Until now, six species: *Scaphoidella acaudata*, *Scaphoidella brevissima*, *Scaphoidella denticlestyla*, *Scaphoidella dietrichi*, *Scaphoidella undosa*, and *Scaphoidella unihamata* are endemic to China and *Scaphoidella stenopaea*, *Scaphoidella undosa*, and *Scaphoidella unihamata*, appear to be widespread. It is highly likely that there are undiscovered species in China.

## Supplementary Material

XML Treatment for Scaphoidella

XML Treatment for Scaphoidella acaudata

XML Treatment for Scaphoidella arboricola

XML Treatment for Scaphoidella brevissima

XML Treatment for Scaphoidella clavatella

XML Treatment for Scaphoidella denticlestyla

XML Treatment for Scaphoidella dietrichi

XML Treatment for Scaphoidella stenopaea

XML Treatment for Scaphoidella undosa

XML Treatment for Scaphoidella unihamata

XML Treatment for Scaphoidella wideaedeaga

XML Treatment for Scaphoidella zhangi

## References

[B1] Anufriev GA (1977) Two new species of Auchenorrhynchous Insects from the Temperate Asia (Homoptera). Reichenbachia 16(21): 211–215.

[B2] Cai P He JH Gu XL (2001) Homoptera: Cicadellidae. In: Wu H Pan CW (Eds) Insects of Tianmushan National Nature Reserve. Science Press, Beijing, China, 132–145 [in Chinese with English summary]

[B3] Dai RH Xing JC Li H (2011) One new species of the genus *Scaphoidella* from China (Hemiptera, Cicadellidae, Deltocephalinae). Journal of Guizhou Normal University (Natural Sciences) 29(3): 1–2 [in Chinese with English summary]

[B4] Dai W Dietrich CH (2011) Review of the Old World leafhopper genus *Scaphoidella* Vilbaste (Hemiptera: Cicadellidae: Deltocephalinae), with description of ten new species from Thailand and Vietnam. Annales de la Société entomologique de France (N.S.) 47(3–4): 457–473. doi: 10.1080/00379271.2011.10697737

[B5] Li ZZ Dai RH Xing JC (2011) Deltocephalinae from China (Hemiptera: Cicadellidae). Popular Science Press, Beijing, China, 336 pp [in Chinese with English summary]

[B6] Li ZZ Kuoh CL (1993) Two new species of the genus *Scaphoideus* from Fujian China (Homoptera: Euscelidae). Journal of Guizhou Agricultural College 12(1): 37–40 [in Chinese with English summary]

[B7] Li ZZ Xing JC (2009) A new species of the genus *Scaphoidella* Vilbaste (Hemiptera: Cicadellidae: Euscelinae) from China. Entomotaxonomia 31(2): 99–101 [in Chinese with English summary]

[B8] Vilbaste J (1968) Systematic treatise of cicadas found on the edge of the coastal regions. Uber die Zikadenfauna des Primorje Gebietes. Valgus, Tallin, 195 pp.

[B9] Viraktamath CA Mohan GS (2004) A revision of the deltocephalinae leafhopper genus *Scaphoideus* (Hemiptera: Cicadellidae) from the Indian subcontinent. Zootaxa 578: 1–48.

[B10] Wang LM Li ZZ (2004) Three new species of the genus *Scaphoideus* (Homoptera: Cicadellidae: Euscelinae) from Yunnan. Entomotaxonomia 26(1): 15–18 [in Chinese with English summary]

[B11] Xing JC Dai RH Li ZZ (2008) A new species of genus *Scaphoidella* from Hainan Province in China (Hemiptera: Cicadellidae: Euscelinae). Sichuan Journal of Zoology 27(6): 963–965. [in Chinese with English summary]

[B12] Xing JC Li ZZ (2010) Hemiptera: Cicadellidae: Eusceline. In: Chen XS Li ZZ Jin DC (Eds) Insects From Mayanghe Landscape.Guizhou Science and Technology Publishing House, Guiyang, China, 132–145 [in Chinese with English summary]

[B13] Zahniser JN Dietrich CH (2013) A review of the tribes of Deltocephalinae (Hemiptera: Auchenorrhyncha: Cicadellidae). European Journal of Taxonomy 45: 1–211. doi: 10.5852/ejt.2013.45

[B14] Zhang YL Dai W (2006) A taxonomic study on the leafhopper genus *Scaphoidella* Vilbaste (Hemiptera: Cicadellidae: Deltocephalinae) from China. Zoological Science 23(10): 843–851. 17116987 10.2108/zsj.23.843

